# Apolipoprotein B and Glycemic Indices in Normoglycemic Adults: Analysis of the National Health and Nutrition Examination Survey, 2007-2016

**DOI:** 10.7759/cureus.85656

**Published:** 2025-06-09

**Authors:** Dayawa D Agoons, Batakeh B. Agoons

**Affiliations:** 1 Department of Medicine, MercyOne North Iowa Medical Center, Mason City, USA; 2 Department of Medicine, NYC Health + Hospitals/Woodhull, New York, USA

**Keywords:** apolipoprotein b, insulin resistance, linear models, prevalence, risk factors

## Abstract

Introduction

Insulin resistance (IR) and pancreatic B-cell dysfunction are fundamental disorders in the pathogenesis of type 2 diabetes. Recent evidence suggests that apolipoprotein B (Apo-B) may be related to the onset of type 2 diabetes. However, the mechanism explaining this association is unclear.

Methods

We analyzed data from 4888 normoglycemic adults pooled from the 2007-2016 National Health and Nutrition Examination Survey (NHANES). Participants were categorized by tertiles of Apo-B, and the main outcome measures were IR and pancreatic β-cell function ascertained by homeostasis model assessment for insulin resistance (HOMA-IR) and homeostasis model assessment for beta cell function (HOMA-β), respectively. Poisson and linear regressions were used to generate prevalence ratios (PRs) and β coefficients for IR and β-cell function, respectively.

Results

Among 4888 participants, the mean Apo-B was 0.85 ± 0.2 g/L, and 532 (10.8%) had IR. After adjusting for demographic variables, the PRs (95% CI) for IR comparing higher tertiles (T2 and T3) with the lowest tertile (T1) of Apo-B were 1.49 (1.19-1.88) and 1.92 (1.54-2.39), respectively. There was a significant increase in log HOMA-β for T2 and T3 compared to T1 of Apo-B, after adjusting for demographic variables (β 0.05 (95% CI: 0.01-0.09) and β 0.15 (95% CI: 0.11-0.19), respectively). Additional adjustment for lifestyle and metabolic variables did not change the significance of these findings. There was a significant graded increase in log HOMA-IR and HOMA-β from T2 to T3 (P for trend <0.001).

Conclusion

Apo-B was associated with increased IR and pancreatic β-cell function in normoglycemic adults independently of traditional risk factors for diabetes. These findings suggest that Apo-B may be associated with the development of glycemic dysregulation.

## Introduction

Insulin resistance and progressive pancreatic β-cell dysfunction are the established fundamental disorders in the pathogenesis of type 2 diabetes [1,2]. Insulin resistance and dyslipidemia increase the risk of cardiovascular disease and its complications in patients with diabetes mellitus [3-6]. Over the last two decades, low-density lipoprotein cholesterol (LDL-C) has been the most popular measurement to assess cardiovascular risk. However, accruing evidence suggests that apolipoprotein B (Apo-B) is a better predictor of cardiovascular disease risk than LDL-C [7-10], since it captures total low-density lipoprotein (LDL), intermediate-density lipoprotein (IDL), very low-density lipoprotein (VLDL), and lipoprotein (a) particle measurements [11]. Interestingly, some guidelines have recommended the addition of Apo-B measurement to the lipid panel assessment in routine clinical practice [12]. Recently, there has been an interest in the role of Apo-B as a predictor of the metabolic syndrome and insulin resistance [13,14]. However, only a few extant studies have evaluated the association of Apo-B with glycemic progression and suggest that Apo-B may be related to an increased risk of incident type 2 diabetes [15,16]. The mechanism by which Apo-B may be related to the onset of type 2 diabetes remains unclear.

Using data from the National Health and Nutrition Examination Survey (NHANES), we sought to evaluate the association of Apo-B with the determinants of incident type 2 diabetes (insulin resistance and pancreatic β-cell function) in normoglycemic adult subjects. We hypothesized that Apo-B would be associated with increased insulin resistance and decreased pancreatic β-cell function in healthy normoglycemic individuals.

## Materials and methods

Study design

This was a cross-sectional study designed to determine the association between Apo-B and insulin resistance and pancreatic beta cell function in normoglycemic adults.

Study setting and participants

The NHANES is a large, nationally representative survey designed to study the health and nutrition status of adults and children in the United States, conducted by the Centers for Disease Control and Prevention. For the current investigation, we pooled NHANES data from 2007 to 2016 and included participants with complete data on Apo-B and fasting insulin. Our inclusion criteria were adult males and females aged 18 years and above, with complete data on Apo-B and fasting insulin. Of the 50,588 eligible participants, there were 35,275 with missing data for Apo-B and 249 for fasting insulin. Since surrogate markers for insulin resistance perform poorly in individuals with type 2 diabetes [17], we excluded participants with self-reported diabetes mellitus or use of blood glucose lowering medications (n = 1952), self-reported prediabetes (n = 725), fasting plasma glucose >100 mg/dL (n = 5028), glycosylated hemoglobin (HbA1c) ≥5.7% (n = 975), and those less than 18 years of age (n = 1496). After applying these exclusions, 4888 adult normoglycemic men and women were included in our analyses.

Variables

Assessment of Apolipoprotein B

In the NHANES, serum Apo-B was directly measured in fasting blood samples by immunoassays. Apo-B immune complexes formed in the serum scatter a beam of light that is proportional to the concentration of Apo-B [18]. Serum Apo-B was measured in grams per liter (g/L).

Assessment of Glycemic Indices

Insulin resistance was ascertained using the homeostasis model assessment for insulin resistance (HOMA-IR) as follows: HOMA-IR = (fasting plasma insulin [µU/mL]) x (fasting plasma glucose [mmol/L]) ÷ 22.5 [19]. HOMA-IR values were not normally distributed and were log-transformed for analysis. Insulin resistance was defined as HOMA-IR ≥75th percentile of its distribution in our population [20].

Pancreatic β-cell function was ascertained using the homeostasis model assessment for beta cell function (HOMA-β) as follows: HOMA-β = (20 x fasting plasma insulin [µU/mL]) / (fasting plasma glucose [mmol/L] - 3.5) [19]. HOMA-β values were not normally distributed and were log-transformed for final analysis.

Assessment of Covariates

Baseline characteristics, including age, sex, race, level of education, body mass index (BMI), smoking status, family history of diabetes, alcohol consumption, and level of physical activity, were obtained with standardized questionnaires. Participants who responded in the affirmative to the question “Do you now smoke cigarettes?” were categorized as current smokers. Alcohol consumption was defined as having ≥12 alcoholic drinks per year. Physical activity was defined as participating in moderate-intensity sports or activities that cause an increase in breathing or heart rate for 10 minutes continuously. Estimated glomerular filtration rate (eGFR) was calculated using the Chronic Kidney Disease Epidemiology Collaboration equation [21].

Data sources

All the necessary information pertaining to the study variables was obtained from the NHANES database. These data were then collected and interpreted.

Sample size

The sample size for this study was not formally collected. All participants meeting the inclusion criteria were recruited. To increase the accuracy of results, we recruited over 10 years’ worth of NHANES data (2007-2016 inclusive). Our inclusion criteria were adult males and females aged 18 years and above, with complete data on Apo-B and fasting insulin.

Data analysis

Participant characteristics were reported across tertiles of Apo-B as mean ± SD and median (IQR) for continuous variables as appropriate, and N (%) for categorical variables. Covariates were compared across tertiles of Apo-B using the analysis of variance, Kruskal-Wallis, and χ2 test as appropriate.

Poisson regression models with robust variance were used to assess the association between Apo-B and insulin resistance, while the association of Apo-B with log-transformed HOMA-IR and HOMA-β was assessed using multivariable linear regression models. Prevalence ratios (PRs) (obtained from Poisson regression models) and β coefficients (obtained from linear regression models) with their 95% confidence intervals (CI) are presented for higher tertiles (T2 and T3) compared to the lowest tertile (T1) of Apo-B.

We conducted adjustments for confounders in a sequential fashion. The first model adjusted for age, sex, race, and BMI (model 1). The second model adjusted for covariates in model 1 in addition to smoking status, alcohol consumption, physical activity, family history of diabetes, and eGFR (model 2). Model 3 further adjusted for serum triglycerides and total cholesterol. All analyses were performed using Stata version 15 (StataCorp, College Station, TX). A two-sided p-value <0.05 was considered statistically significant.

Ethical consideration

As this study used data from a publicly available dataset, ethical clearance was not required. The NHANES contains de-identified participant data.

## Results

Descriptive data of study participants

Table 1 shows the baseline characteristics of participants by tertiles of Apo-B. The mean age of the study population was 38.9 ± 16.7 years, and 2891 (59.1%) were women. The mean Apo-B of the study population was 0.85 ± 0.2 g/L. The mean Apo-B was 0.87 ± 0.2 g/L in men (n = 1997) and 0.84 ± 0.2 g/L in women (n = 2891). Compared to the lowest Apo-B tertile, participants in the highest tertile were older, had a higher BMI, waist circumference, fasting plasma glucose, fasting serum insulin, systolic and diastolic blood pressure, and were more likely to be current smokers.

**Table 1:** Characteristics of participants by tertiles of apolipoprotein B (Apo-B). Data are presented as mean ± SD for continuous variables and N (%) for categorical variables. * Alcohol consumption was defined as ≥12 alcoholic drinks/year. ^α^ Data presented as median (interquartile range). P-value <0.05 was considered statistically significant. P-values were generated using ANOVA when comparing continuous variables and the chi-square test when comparing categorical variables. Test statistic value refers to the numerical value of the statistical test used to generate the p-value (^γ^ F-value for ANOVA and ^µ^ chi-square value for chi-square test). T1: Apo-B first tertile; T2: Apo-B second tertile; T3: Apo-B third tertile; SBP: systolic blood pressure; DBP: diastolic blood pressure; eGFR: estimated glomerular filtration rate; HOMA-IR: homeostasis model of assessment for insulin resistance; HOMA-β: homeostasis model of assessment for pancreatic beta cell function.

	T1 (<0.76)	T2 (0.76-0.97)	T3 (>0.97)		
Characteristics	(n = 1,815)	(n = 1,723)	(n = 1,350)	Test statistic value ^γ, µ^	P-value
Age, years	33.7 ± 15.6	40.6 ± 16.8	43.7 ± 16.2	160.8	<0.001
Women, N (%)	1131 (62.3)	1032 (59.9)	728 (53.9)	23.1	<0.001
Race, N (%)				34.4	<0.001
Non-Hispanic White	742 (40.9)	786 (45.6)	629 (46.6)		
Non-Hispanic Black	405 (22.3)	294 (17.2)	198 (14.7)		
Education, N (%)				23.7	0.001
Less than high school	265 (17.3)	324 (20.2)	296 (22.8)		
High school graduate	289 (18.9)	321 (20.1)	286 (21.9)		
Attended college or higher	973 (63.6)	956 (59.7)	719 (55.3)		
BMI, kg/m^2^	25.2 ± 5.8	26.9 ± 6.0	28.3 ± 5.7	112.5	<0.001
Waist circumference, cm	86.5 ± 13.6	92.1 ± 14.5	97.2 ± 13.6	221.1	<0.001
Current smoker, N (%)	324 (58.4)	335 (53.4)	277 (50.2)	7.6	0.022
Alcohol consumption*, N (%)	1114 (72.5)	1100 (73.7)	902 (73.8)	0.7	0.699
SBP, mmHg	113.2 ± 13.5	116.4 ± 14.8	119.8 ± 16.4	69.3	<0.001
DBP, mmHg	64.9 ± 11.1	67.8 ± 11.2	69.4 ± 11.9	59.3	<0.001
eGFR, ml/min/1.73 m^2^	109.9 ± 21.9	103.5 ± 21.8	100.7 ± 21.9	75.1	<0.001
Log HOMA-IR	0.43 ± 0.6	0.58 ± 0.6	0.73 ± 0.6	80.5	<0.001
Log HOMA-β	4.5 ± 0.6	4.6 ± 0.7	4.7 ± 0.6	38.5	<0.001
Fasting plasma glucose, mg/dL	90.1 ± 6.2	91.3 ± 6.2	92.0 ± 5.9	38.3	<0.001
Fasting insulin, µU/ml^α^	6.9 (4.6-10.1)	7.6 (5.2-11.9)	9.1 (6.9-13.8)	44.6	<0.001

Outcome data

Assessment of Apo-B With Insulin Resistance

Of the 4888 participants, 532 (10.8%) had insulin resistance as defined by a HOMA-IR ≥75th percentile of its distribution in our population.

Table 2 shows the association of Apo-B with HOMA-IR and insulin resistance. Higher tertiles of Apo-B were associated with an increase in the prevalence of insulin resistance compared to the lowest tertile. After adjustment for age, sex, race, and BMI, prevalent insulin resistance was 1.49 times greater in tertile 2 of Apo-B than tertile 1 (PR: 1.49 (95% CI: 1.19-1.88), P < 0.001) and 1.92 times greater in tertile 3 compared to tertile 1 (PR: 1.92 (95% CI: 1.54-2.39), P < 0.001). Also, the prevalence of insulin resistance significantly increased with the higher tertile of Apo-B (P of trend <0.001) (Table 2, model 1). Additional adjustment for smoking, alcohol consumption, physical activity, family history of diabetes, and eGFR (tertile 2 vs. tertile 1: PR: 1.55, 95% CI: 1.18-2.04, p = 0.002; tertile 3 vs. tertile 1: PR: 2.05, 95% CI: 1.58-2.66, p < 0.001) (Table 2, model 2), and further adjustment for total cholesterol and triglycerides (tertile 2 vs. tertile 1: PR: 1.92, 95% CI: 1.43-2.58, p < 0.001; tertile 3 vs. tertile 1: PR: 3.04, 95% CI: 2.09-4.39, p < 0.001) (Table 2, model 3) did not change the significance of these results. The results were similar for log HOMA-IR. The second tertile of Apo-B had a 0.14-unit increase in log HOMA-IR compared to the first tertile (β coefficient: 0.14, 95% CI: 0.09-0.20, p < 0.001), and the third tertile had a 0.29-unit increase in log HOMA-IR when compared to the first tertile (β coefficient: 0.29, 95% CI: 0.19-0.38, p < 0.001) (Table 3, model 3).

**Table 2:** Association of apolipoprotein B (Apo-B) with insulin resistance*. * Insulin resistance is defined as a HOMA-IR ≥75th percentile of its distribution in our population. Model 1 adjusted for age, sex, race, and BMI. Model 2 adjusted for variables in model 1 with additional adjustment for smoking, alcohol consumption, physical activity, family history of diabetes, and estimated GFR. Model 3 included variables in model 2 with further adjustment for total cholesterol and triglycerides. P-values were generated from multivariate Poisson regression. PR: prevalence ratio; T1: Apo-B tertile 1; T2: Apo-B tertile 2; T3: Apo-B tertile 3.

Tertiles of Apo-B, g/L	Model 1		Model 2		Model 3	
	PR (95% CI)	P-value	PR (95% CI)	P-value	PR (95% CI)	P-value
T1: <0.76	1 (Reference)	…	1 (Reference)	…	1 (Reference)	…
T2: 0.76-0.97	1.49 (1.19, 1.88)	<0.001	1.55 (1.18, 2.04)	0.002	1.92 (1.43, 2.58)	<0.001
T3: >0.97	1.92 (1.54, 2.39)	<0.001	2.05 (1.58, 2.66)	<0.001	3.04 (2.09, 4.39)	<0.001
P of trend		<0.001		<0.001		<0.001

**Table 3:** Association of apolipoprotein B (Apo-B) with insulin resistance (log HOMA-IR). Model 1 adjusted for age, sex, race, and BMI. Model 2 adjusted for variables in model 1 with additional adjustment for smoking, alcohol consumption, physical activity, family history of diabetes, and eGFR. Model 3 included variables in model 2 with further adjustment for total cholesterol and triglycerides. P-values were generated from multivariate linear regression. T1: Apo-B tertile 1; T2: Apo-B tertile 2; T3: Apo-B tertile 3; HOMA-IR: homeostasis model assessment for insulin resistance; eGFR: estimated glomerular filtration rate.

Tertiles of Apo-B, g/L	Model 1		Model 2		Model 3	
	β (95% CI)	P-value	β (95% CI)	P-value	β (95% CI)	P-value
T1: <0.76	1 (Reference)	…	1 (Reference)	…	1 (Reference)	…
T2: 0.76-0.97	0.09 (0.05, 0.13)	<0.001	0.09 (0.04, 0.13)	<0.001	0.14 (0.09, 0.20)	<0.001
T3: >0.97	0.19 (0.14, 0.23)	<0.001	0.18 (0.14, 0.23)	<0.001	0.29 (0.19, 0.38)	<0.001
P of trend		<0.001		<0.001		<0.001

Assessment of Apo-B With Pancreatic β-Cell Function

Table 4 shows the association between Apo-B and pancreatic β-cell function as assessed by HOMA-β (log transformed for analysis). In multivariate regression models, there was a 0.05-unit increase in log HOMA-β for the second Apo-B tertile compared to the first tertile (β coefficient: 0.05 (95% CI: 0.01-0.09), p < 0.001), while there was a 0.15-unit increase in log HOMA-β for the third tertile when compared to the first tertile (β coefficient: 0.15 (95% CI: 0.11-0.19), p < 0.001) (Table 4, model 1). There was a graded increase in log HOMA-β from the second to the third tertile of Apo-B (P of trend <0.001). After full adjustment for confounders, the significance of the results remained the same (Table 4, model 3).

**Table 4:** Association of apolipoprotein B (Apo-B) with pancreatic beta cell function (log HOMA-β). Model 1 adjusted for age, sex, race, and BMI. Model 2 adjusted for variables in model 1 with additional adjustment for smoking, alcohol consumption, physical activity, family history of diabetes, and eGFR. Model 3 included variables in model 2 with further adjustment for total cholesterol and triglycerides. P-values were generated from multivariate linear regression. T1: Apo-B tertile 1; T2: Apo-B tertile 2; T3: Apo-B tertile 3; HOMA-β: homeostasis model assessment for beta cell function; eGFR: estimated glomerular filtration rate.

Tertiles of Apo-B, g/L	Model 1		Model 2		Model 3	
	β (95% CI)	P-value	β (95% CI)	P-value	β (95% CI)	P-value
T1: <0.76	1 (Reference)	…	1 (Reference)	…	1 (Reference)	…
T2: 0.76-0.97	0.05 (0.01, 0.09)	0.041	0.04 (-0.01, 0.09)	0.081	0.09 (0.03, 0.15)	0.003
T3: >0.97	0.15 (0.11, 0.19	<0.001	0.15 (0.10, 0.19)	<0.001	0.23 (0.15, 0.31)	<0.001
P of trend		<0.001		<0.001		<0.001

## Discussion

We evaluated the association between Apo-B and glycemic indices (insulin resistance and pancreatic β-cell function) in a large sample of healthy normoglycemic individuals. We found that Apo-B was associated with increased insulin resistance and pancreatic β-cell function independently of traditional risk factors for diabetes mellitus. The associations tended to be stronger for higher tertiles of Apo-B both for insulin resistance and β-cell function. These findings suggest that Apo-B may be involved in the development of glycemic dysregulation.

Our study adds to the current literature as it explores an assessment of the association between Apo-B and various aspects of glucose dysregulation (insulin resistance and insulin secretory function) in normoglycemic individuals. Our findings are similar to those of previous studies that have examined the relationship of Apo-B with insulin resistance in other populations [14,22-24]. Sung et al. showed that Apo-B was positively correlated with insulin resistance in normoglycemic Koreans [25]. However, the authors did not explore an association between Apo-B and pancreatic β-cell function.

Our findings are also supported by recent population-based studies. A study from Korea found that Apo-B was significantly associated with insulin resistance even after adjusting for conventional metabolic risk factors, highlighting its utility in identifying individuals at metabolic risk despite normal glucose levels [26]. Similarly, in a family practice cohort, Apo-B levels were found to increase proportionally with HOMA-IR and HOMA-β, reinforcing its role as a dual marker of insulin resistance and β-cell compensation [27]. These findings are consistent with our results and suggest that Apo-B may serve as an accessible biomarker in primary prevention strategies for metabolic disorders.

A few mechanisms have been proposed to explain the relationship between Apo-B and increased insulin resistance. Laboratory-based studies have suggested a close association between hepatic VLDL-Apo B overproduction and insulin resistance, which increases the activity of protein-tyrosine phosphatase-1B (PTP-1B) and facilitates secretion of Apo-B-containing lipoprotein particles [28,29]. Additionally, the accumulation of lipids within muscle and liver cells triggers protein kinase C activation, which impairs insulin signaling [30]. The increased β-cell function we observed in our study could be due to a compensatory reaction of the endocrine pancreas to increased insulin resistance, a phenomenon well known to characterize the initial phase of the glucose dysregulation process [31].

Our findings could have some clinical implications. Apo-B could potentially serve as an indicator for the risk of prevalent insulin resistance and glycemic dysregulation. This could allow for the development of targeted interventions aimed at lowering plasma Apo-B and potentially decreasing the risk of prevalent insulin resistance. The development of these interventions could offer a dual benefit to patients as Apo-B has also been identified as a strong risk factor for atherosclerosis and cardiovascular disease [12,32]. Furthermore, this raises the possibility of exploring the effects of lipid-lowering therapies such as statins and proprotein convertase subtilisin/kexin type 9 (PCSK9) inhibitors, which have also been shown to lower Apo-B. These therapies potentially could inhibit the development of insulin resistance in normoglycemic individuals and stymie the glucose dysregulation process in this population.

Our study has limitations that should be acknowledged. First, this was a cross-sectional study and therefore it is subject to the issues of unmeasured, residual confounding, and reverse causation. Hence, this limits our ability to establish causality between Apo-B and the glycemic indices we evaluated. Second, we excluded participants from our study with missing data on key variables, which could introduce bias to our results. Third, we ascertained insulin resistance and β-cell function using the homeostasis model assessment (HOMA) instead of the gold standard euglycemic clamp. However, the HOMA has been validated and provides estimates of insulin sensitivity that correlate with the euglycemic clamp.

Despite these limitations, our study has multiple strengths. First, we used a large, diverse, and nationally representative sample of participants. Second, none of our study participants were on lipid-lowering medications such as statins, which affect β-cell function and decrease insulin secretion. Such exclusion improved the robustness of our results. Furthermore, Apo-B was measured using standardized methods, and measurements were less influenced by body weight since the mean BMI of our population was <30 kg/m^2^.

## Conclusions

In a large sample of normoglycemic adult men and women, Apo-B was associated with increased insulin resistance and pancreatic β-cell function independently of traditional risk factors for diabetes. These results suggest that Apo-B may be associated with the development of glycemic dysregulation. Prospective studies are needed to evaluate the relationship of Apo-B with incident insulin resistance and glycemic progression in normoglycemic subjects.

## Appendices

What is known about this topic?

A few studies have suggested an association between apolipoprotein B and type 2 diabetes. However, the mechanism of this association is unknown. 

What This Study Adds?

Apolipoprotein B was associated with increased insulin resistance and pancreatic beta cell function in normoglycemic men and women.

The association tended to be stronger for higher tertiles of apolipoprotein B, both for insulin resistance and pancreatic beta cell function.
